# Living with obsessive-compulsive disorder (OCD): a South African narrative

**DOI:** 10.1186/s13033-018-0253-8

**Published:** 2018-12-01

**Authors:** Kirsten Celeste Kohler, Bronwynè Jo’sean Coetzee, Christine Lochner

**Affiliations:** 1 Department of Psychology, Stellenbosch University, Stellenbosch, South Africa; 2 SU/UCT MRC Unit on Risk and Resilience in Mental Disorders, Department of Psychiatry, Stellenbosch University, Stellenbosch, South Africa

**Keywords:** Obsessive–compulsive disorder, Daily functioning, Quality of life, Qualitative, Thematic analysis

## Abstract

**Background:**

Obsessive–compulsive disorder (OCD) is a highly prevalent and debilitating psychiatric disorder known to interfere with several life domains. Yet little is known about the subjective experiences of living with OCD amongst South Africans and more so, the ways in which it impacts daily functioning and quality of life (QOL).

**Methods:**

The aim of this study was to explore daily functioning and QOL among South African adults living with OCD. Qualitative semi-structured interviews were conducted with 20 adults with a primary diagnosis of OCD. We used ATLAS.ti v7 to analyse the data, thematically. The study was conducted at the SU/UCT MRC Unit on Risk and Resilience in Mental Disorders in South Africa.

**Results:**

Three key themes were identified namely, (1) realisation of OCD, (2) disruptions to daily life and (3) managing the disruptions to daily life. Participants recounted their earliest recollections of OCD, the instances when they recognised something was wrong and ways in which they came to terms with their OCD. Disruptions to daily life included poor sleep quality, inability to enjoy leisure activities which impacted on socialisation and impairment in school/work performance. Perceived social support from family members, friends and colleagues were invaluable to helping participants manage these disruptions. Further, strategies such as self-talk, diary-keeping and humour helped them cope.

**Conclusion:**

While some individuals with OCD have found ways to cope with and accept having OCD, all participants perceived their QOL to be significantly reduced and their functioning impaired due to the condition, on multiple levels. The importance of acceptance in OCD ties in with research on the potential value of Acceptance and Commitment Therapy, which could form an adjunct to more conventional techniques such as Cognitive-Behavioural Therapy. The themes emanating from this study can be used to help clinicians better understand what treatment works best for patients with OCD—and whether this treatment be focused on the individual or together with close members of their microsystem, such as spouses/partners. Further these findings may potentially help to improve access, affordability and the quality of life of South Africans living with OCD from various income backgrounds.

## Background

Obsessive–compulsive disorder (OCD), a highly prevalent psychiatric condition, is recognised as one of the 10 most disabling conditions given its impact on functioning and quality of life (QOL) [1, 2]. OCD is characterized by recurrent and intrusive thoughts and images (obsessions) and/or repetitive behaviours (known as compulsions) [3].

There is a substantial body of quantitative evidence to suggest that OCD has a large negative influence on the daily activities and QOL of individuals living with the disorder [4–9]. However, there are some discrepancies in findings on the ways OCD affects life. For example, amongst an Indian OCD sample, the psychological and social domains of QOL were more affected than other domains [6]. In contrast, evidence from a South African sample of OCD patients suggested impairment in family functioning but not in other domains [8].

Qualitative studies offer a way in which these discrepancies may potentially be addressed, providing additional insights into how individuals experience disruptions in the various domains of functioning due to OCD. Improvements in understanding may ultimately lead to better management of these patients [10–16]. For example, some insights into the experiences of OCD are seen in a qualitative study from India [17]. In this study participants articulated that their obsessions and compulsions often made it difficult to maintain a connection with others which limited their access to emotional support and often lead to feelings of hopelessness and powerlessness, and impacted on self-esteem [10, 11, 13, 14, 17]. Findings such as these begin to provide deeper insights into individuals’ experiences of the impact of OCD on their lives.

At the time of this writing, no publications on qualitative studies on OCD amongst South African individuals were available. Locally, studies on OCD that are available have mainly focused on the functional impairment aspects of OCD, quantitatively [8, 18–20]. The OCD symptom profile may look similar amongst individuals living with the disorder globally; however, it may be that the broader social, political and economic factors influence and shape the perceived experience of the condition differently amongst those living in resource-limited and thus vulnerable settings like South Africa. Accordingly, in this study, we used qualitative methods to explore the experiences of and ways in which OCD influences the daily life and functioning of South African adults living with OCD.

### Theoretical framework

Globally, mental health researchers increasingly value the importance of exploring mental health holistically, through biological, psychological and sociocultural perspectives [21]. Much mental health research has been orientated towards understanding the individual, often through a biomedical lens, with little focus on the influences of the broader context within which the individual lives and how this may impact on functioning [21]. For example, various individual models exists to understand OCD [12, 22–24]. More recent models of OCD—which go beyond the better known cognitive models of OCD [25, 26], is the inference-based approach (IBA) and the autogenous-reactive model (AR). The IBA is a cognitive model of OCD [22, 23] which posits that an individual’s fear of the self is core to various obsessions experienced. Further, the AR model of OCD [24] posits that an individual’s obsessions are triggered either internally (within the individual—e.g. through memories) or externally (i.e. triggered by external stimuli—e.g. accidents/fires) [12].

In this study, we were interested in going beyond individual factors associated with OCD and exploring the ways in which OCD impacts various life domains where interpersonal relationships are key. To this end, we used concepts associated with Ecological Systems Theory (EST), as laid out by Bronfenbrenner [27, 28] to guide explanations of the influence of OCD on daily functioning. In keeping with EST, the individual functions within a system of nested environments, and each of these environments is connected to one another in a bi-directional way. As such, these systems function together to affect an individual’s development such as their relationships, emotions, behaviour, and general functioning. In EST these systems include (1) the microsystem—i.e. the relationship between the individual and those within their immediate environments such as members of their family and work colleagues; (2) the mesosystem—which refers to the interrelations between two or more microsystems surrounding the individual such as the relationship between the individual’s family and workplace, and family and peers; (3) the exo system—i.e. the link between two or more systems with whom the individual has indirect interaction with, but has an effect on the individual such as workplace policies; (4) the macro system—i.e. the social factors that affect the individual’s life, such as the individual’s ideology, and (5) the chronosystem—which takes into account the changes in an individual’s life over time [27, 29]. Our study is predominantly concerned with the microsystem of the individual’s life. As such, we present findings on the impact of OCD on the individual and his/her immediate interpersonal relationships in multiple settings.

## Methods

### Design

We used an exploratory qualitative research design and gathered data from individuals with a primary diagnosis of OCD using both qualitative and quantitative data collection methods. OCD was diagnosed using the Structured Clinical Interview for DSM disorders—SCID-I/P [30].

### Setting

The present study was conducted at the Medical Research Council’s (MRC) Unit on Risk and Resilience in Mental Disorders. This is a cross-university research unit located at the Department of Psychiatry at Stellenbosch University (SU) and the Department of Psychiatry and Mental Health at the University of Cape Town (UCT), South Africa. This unit follows in the footsteps of the MRC Unit on Anxiety and Stress Disorders that was initiated at SU in 1997. Both are known for work on psychiatric genetics, psychiatric brain imaging, basic neuroscience, and mental health promotion. Foci include work on animal models, on clinical research, and on public health aspects. This study was embedded in a larger quantitative study focused on the phenomenology as well as the pharmacological, neurological and genetic underpinnings of OCD that was initiated to better understand the cognitive-affective processes in adult OCD patients [31, 32]. The aims of the larger study (still ongoing) are to collect neuroimaging data (via structural and functional MRI), clinical data (via self-report and clinician administered questionnaires), and genetic data (obtained via blood samples), to enable South African researchers to make contributions to international OCD research consortia and stimulate the growth of disciplines and interdisciplinarity. In addition, it is aimed that this larger project renders new knowledge on genotype-interaction effects on key brain structures in OCD. Findings related to these aspects of the larger study are reported elsewhere [33–35]. The present study sought to expand on the quantitative data collected as part of the larger study by including patient perspectives on the impact of OCD in their everyday lives.

### Participants and procedure

A subset of participants from the larger study were consecutively selected to receive an invitation via e-mail to be interviewed. Eligible participants were 18 years or older, with a primary diagnosis of OCD. Given that participants were recruited consecutively from the database of the larger study, no specific attempts were made to purposively recruit for gender or socioeconomic status. Recruitment of participants continued until no more new information emerged from the interviews, that is until we reached data saturation [36]. The primary diagnosis of OCD was confirmed using DSM-IV criteria. Participants were interviewed face-to face if they responded to an email invitation confirming an interest in taking part. Interviews were conducted in either English or Afrikaans, in the participants’ first language, and guided by a semi-structured interview schedule that contained a series of open-ended questions. Interviews generally lasted between 30 and 60 min. These interviews were the first contact that the interviewer (KK) had with the participants. The themes covered in the interview were (1) his/her earliest recollection of OCD symptoms, (2) his/her daily activity experiences, and (3) the coping strategies that were used over time.

Participants completed a demographic questionnaire and the Florida Obsessive–Compulsive Inventory (FOCI) [37] to measure OCD symptom severity before the start of the interview. Score categories included, mild, moderate, severe, and extreme. Additionally, a clinician-administered scale, the Yale-Brown Obsessive Compulsive Scale (Y-BOCS) [38], was also used to assess OCD severity. Score categories also included, mild, moderate, severe, and extreme. These two severity scales (FOCI and Y-BOCS) were included to provide a better understanding of the illness profile of the participants. Trustworthiness of the qualitative data was ensured by clarifying participant responses for meaning using paraphrasing techniques.

### Ethics approval

This study received approval as an amendment to the larger study by the Health Research Ethics Committee (HREC) at Stellenbosch University (M07/05/019). All participants gave written informed consent. Prior to signing the consent forms, participants were informed that participation in the present study was a once-off semi-structured interview. Participants were also informed that their participation was entirely voluntary and that they were free to withdraw from the study at any point without consequence. Further, participants were informed that they could refuse to answer any questions. Further, participants were assured that their information would be kept strictly confidential and only accessible to the student (KK), and her two supervisors (BJC & CL). Participants were informed that their data would be completely anonymized in any publications emanating from this work, and that the data will be stored securely on the password-protected computers of the student and her supervisors. Participants each received a travel voucher to reimburse them for the costs of their journey to and from the research unit.

### Data analysis

Interviews were digitally recorded (with permission from the participants) and transcribed verbatim. The transcripts were thematically analysed (TA) using ATLAS.ti version 7, a computer supported qualitative data analysis software. Analysis of the transcripts followed a six stage approach to data analysis, i.e.: (1) familiarisation with the data, (2) coding relevant extracts of the data and generating an initial code list, (3) refining the codes and generating themes, (4) reviewing each theme generated in step 3, (5) refining, describing, and allocating descriptive labels to each theme, and (6) presenting the results of the data analysis [39]. In qualitative research, themes can be generated either inductively (from the data alone), deductively (informed by theory), or via a combination of the two [40]. In this study, we used a combination of the two methods. As such, EST provided a useful lens through which to interpret the inductively generated themes across the various systems in which the individual functions [41]. Pseudonyms have been used to protect participants’ identities.

## Results

### Participants’ characteristics

As depicted in Table 1, we interviewed 20 adults (mean age 45.65) with a primary diagnosis of OCD, the majority of whom were female (75%). A large proportion of participants were married (60%), and living with other adults and/or children (40%). Half of our participants (50%) graduated from university/college and most (60%) were employed either full-time or part-time. A large proportion of participants (70%) reported earning R15,000 (~ $105USD) or more per month. Illness severity (Y-BOCS- and FOCI-) scores ranged from 8 to 34 (M = 22.4 and SD = 6.62) and 5–18 (M = 10.85 and SD = 4.11), respectively, suggesting moderate severity of OCD on average over the past 7 days.

**Table 1:** Demographics table

Characteristics	n = 20	%
*Age [mean (standard deviation) in years]*	45.65 (13.82)	
*Gender*		
Male	5	25
Female	15	75
*Marital status*		
Single	6	30
Married	12	60
Divorced	2	10
*Living situation*		
Live alone	5	25
Live with other adult(s), no children	7	35
Live with other adults and children	8	40
*Highest education level*		
Completed high school/matric	7	35
Attended university/college but did not graduate	3	15
Graduated from university/college	10	50
*Current work situation*		
Employed full time	10	50
Employed part time	2	10
Student	2	10
Unemployed	1	5
Homemaker	2	10
Retired	3	15
*Family income*		
R2501–R5000	2	10
R5001–R10,000	2	10
R10,001–R15,000	1	5
R15,001 and above	14	70
Unknown	1	5
*FOCI scores*	10.85 (4.11)	
*Y-BOCS scores*	22.4 (6.62)	

### Overview of themes and sub-themes

Table 2 below provides an overview of the themes and sub-themes that were identified following thematic analysis of the data. The main themes centred on participants’ realisations of having OCD, the disruptions caused and the ways in which these were perceived and addressed.

**Table 2:** Themes and sub-themes following thematic analysis

Themes	Sub-themes
Realisation of OCD	Feeling more alike than different
	Recognising something’s wrong
	Coming to terms with OCD
Disruptions to daily life	Disruptions in sleep and rest
	Disruptions to leisure activities and hobbies
	Disruptions to productivity
Managing the disruptions to daily life	Perceived social support
	Coping strategies

#### Theme 1: Realisation of OCD

The theme ‘Realisation of OCD’ portrays participants’ journey with OCD, from their earliest recollections of their symptoms typical of the disorder, to some of the difficulties associated with coming to terms with OCD across their lifetime. Overall, this theme portrays the impact of OCD on the self.

##### Subtheme 1.1. Feeling more alike than different

Participants recounted their early experiences of their obsessive–compulsive symptomatology and explained that ‘back then’ they did not recognise their symptoms as irrational or excessive or as indicative of having a psychiatric condition. Rather, participants considered their thoughts and behaviours to be similar to those of other people, and a natural part of their personality. One participant reported:

*I thought that it was normal to be this way because I have been this way since I was little (Sally, 62*-*year*-*old female).*

Another participant recounted:

*My earliest memory was when I was bout eleven or twelve years old, I was in standard three. I only realised this later on in life but if I look back, my mother used to come into my room and say to me, why have you got so much homework, why are you spending so much time in your room? Meanwhile what I was doing was busy organising my stationery and my books and rearranging them, and packing them neatly, just, you know, doing these things over and over and over again and telling my mother I was busy with my homework. Meanwhile I wasn’t actually achieving much but that’s the first time I realised, well I didn’t realise at that age I had OCD but looking back I think that was the earliest time that I can remember having OCD (Angela, a 40*-*year*-*old female).*

##### Subtheme 1.2. Recognising something’s wrong

Both Sally and Angela recognized the pathological nature of their symptoms only later in life. Their externalised behaviours (compulsions) alerted members in their microsystem [e.g. parents (in childhood) and children and spouses (in adulthood)] that they needed help. One participant recounted:

*I think my mom recognized ‘oh goodness, there is something wrong with this child!’ Yes, she took me to various doctors… and I got diagnosed quite quickly (Rezaan, a 23*-*year*-*old female*).

Another participant stated:

*My children told me that it’s not nice to come and visit me. I could not sit still, I still cannot sit still! […]. And worst of all, I did not realise that it was abnormal (Lucia, a 55*-*year*-*old female).*

##### Subtheme 1.3. Coming to terms with OCD

Participants shared mixed emotions about having OCD; some continued to be angry, sad and resentful whereas others proved more accepting towards the diagnosis. One participant stated:

*… I’ve had enough of it, like I’ve been dealing with it since junior school and I hate my thoughts, I hate my little tics, so some days maybe a teensy bit satisfied but overall I’m not satisfied, no (Kelsey, a 31*-*year*-*old female).*

Similarly Stuart reported:

*I’m angry, I’m disappointed and I’m sad that I had to have gone through it but what can one do. One can’t go back in time, one can’t …but yes it is sad. It’s sad that I can’t have happy memories of overseas trips, it’s sad that I can’t have happy memories of school, it’s sad that I can’t have happy memories of varsity, it’s sad that I can’t have happy memories of my first working life (Stuart, a 47*-*year*-*old male).*

Both Kelsey and Stuart described a resentment towards their OC symptoms and the intrusive nature of their OCD over time. This persistent intrusiveness lead to irritability, anger, sadness, discomfort, disgust and embarrassment. However, unlike Kelsey and Stuart, Derrick explained that he was able to accept OCD as part of his life and developed ways to improve his self-image, despite having OCD:

*I’ve learned to accept myself and I’ve learned to focus on the things on my, you know my…positive things, the good things. So and I started dressing in a way that I feel more comfortable and in developing a style that I like (Derrick, a 58*-*year*-*old male).*

Also claiming acceptance of her condition, another participant stated:

*So it’s definitely gotten a lot better I just don’t think it will ever go away you know, and I think I’ve accepted that (Sasha, a 37*-*year*-*old female).*

#### Theme 2: Disruptions to daily life

The theme ‘Disruptions to daily life’ provides an account of the important disruptions that took place in participants’ lives due to OCD. These include the impact of OCD on activities of daily living such as sleep, work and leisure, as well as the ability to establish and maintain interpersonal relationships with key members of the individual’s microsystem.

##### Subtheme 2.1. Disruption to sleep and rest

Participants reported that the persistent nature of their intrusive thoughts (obsessions) and the resulting anxiety lead to difficulties sleeping and resting. Participants’ lack of sleep meant that they were tired often, and not emotionally or physically available for active engagement with their family and friends.

One participant stated,

*I couldn’t switch off, I would go a whole night worrying about the same thing and tomorrow morning wake up if I slept. Some nights I wouldn’t sleep and not worry about the same thing (Rheinart, a 55*-*year*-*old male).*

Another participant recounted:

*I missed a lot of sleep, because my ritual before going to bed was obviously in the bathroom, and I would be in the bathroom doing my ritual over and over and over until I was sobbing on the floor in tiredness (Wendy, a 26*-*year*-*old female).*

##### Subtheme 2.2. Disruption to leisure activities and hobbies

Participants also reported avoiding activities they enjoyed as many of these triggered symptoms of OCD. These included reading, watching TV, going on holiday, exercising, and participating in sport and hobbies. Not only did the OCD affect participation in these activities, it also impacted on participants’ ability to engage socially. As one participant stated:

*…myself and a friend of mine last year we went and did a trip up to the Northern Cape and down the West Coast just taking photos of cemeteries, and he would’ve wanted to go for probably maybe 2* *weeks, and I said no 4* *days. I can’t be away from my house longer than 4* *days. So he had to change that trip to 4* *days because that was already the limit of me being away from the house… that the house is not being checked (Marcy, a 59*-*year*-*old female).*

Similarly the following participant explained:

*It’s hard for me to relax, my mind doesn’t stop, my mind is in constant worry, in anticipation of bad things. Obviously yes, there are times when I can go out with some friends and my mind will be there, but a lot of the time it interferes with me trying to relax. Always on the edge of worrying about saying something wrong, or obsessing about something or whatever (Wendy, a 26*-*year*-*old female).*

##### Subtheme 2.3. Disruptions to productivity

Participants reported both positive and negative aspects of the influence of their OCD on productivity. On the one hand, there were those who experienced a decline in school and work performance and attendance, resulting in poor productivity. One participant reported:

*There are days that I’ve spent 8* *hours at work and not been able to do a single stitch of work because of the routines that I’ve had to do with OCD, and get to work and you’ve gotta check your e*-*mails. Then all the e*-*mails have to be in bold and then you, and then some of them are unbolded and then no okay it must all be unbolded then you unbold them and they’ve gotta be colour*-*coded. Then they’ve gotta be this then they’ve gotta be that […] and so there’ve been days that I haven’t been able to do any work (Kelsey, a 31*-*year*-*old female).*

Similarly, one participant recounted the negative impact of OCD on academic performance:

*At varsity I couldn’t function at all, because the only way it would make any sense for me is if I took down every single word including “a’s” and “the “s’s” and whatever that that lecturer said, and I took it down neatly that I could be proud of my notes and they were neatly and whatever. That was impossible at Varsity, so I just stopped going to lectures and I used to bum notes off my other people and whatever (Rezaan, a 23*-*year*-*old female).*

On the other hand, there were those who reported that OCD did not impact much on their academic or work performance and sometimes even improved their occupational performance:

*I think I did better than I would otherwise have done, because I pushed myself so much, I was my own biggest competition. It made me perform better (Judy, a 33*-*year*-*old female).*

#### Theme 3: Managing the disruptions to daily life

The theme ‘Managing the disruptions to daily life’ provides an account of the sources of support and the coping strategies that were implemented to deal with the impact of OCD.

##### Subtheme 3.1. Coping strategies

Over time participants found personal ways of adjusting to, coping with and accepting their condition. Strategies included (1) self-talk, (2) keeping a diary of events and situations that may trigger OCD, (3) using humour and other ways of distraction (such as working, taking part in sport, talking to others or using an elastic band), and (4) comfort eating or drinking alcohol. For example one participant explained:

*I’m good now at telling myself to just stop, just put it down just stop because this is not fun anymore uhm (Sasha, a 37*-*year*-*old female).*

Another participant articulated:

*So I learned a lot of methods to write it down and it also works to read it to yourself over and over and over and over and over again until you realize how irrational it is (Judy, a 33*-*year*-*old female).*

Judy went on state that:

*…And I put a rubber band on my arm/wrist and I shoot myself with dizziness if I get this stuff in my head… (Judy).*

##### Subtheme 3.2. Perceived social support

Participants were ambivalent about the support that they received. Some were positive believing that their significant others assisted in improving their QOL whereas others were critical about the ways in which they were treated. For example, some participants spoke of various individuals in their lives who provided solid support to them, and who were invaluable to managing the impact of OCD on their lives. They considered family members as important sources of support, as they provided physical (e.g. taking part in rituals), emotional (e.g. providing constant reassurance), and financial support to participants. One participant stated:

*…when I was diagnosed with OCD they were very supportive, very very supportive. In terms of financially supporting my therapy, financially supporting my education, and emotionally supporting me* etc*. (Stuart).*

Another participant explained how his children’s involvement in his rituals provided reassurance:

*I would ask my children…, they all drive*- *they (are) all big and I would ask them to ride to a specific corner and go and check if there is not a cyclist going there. In the end I think they might have done it once or twice (Rheinart, a 55*-*year*-*old male).*

Participants also spoke of the strong support they received from their close friends, their healthcare providers, colleagues at work and support group members. Humour, in particular, was perceived as a supportive gesture and helped some participants to function and feel accepted—socially and at work—despite OCD. One participant mentioned how her and her friends would humour her situation,

*…For example, a story like Dr. Monk, who is obsessive*–*compulsive, then I always said to them, “I’m Mr. Monk, “then they laugh, then they click and say,” Yes, yes, now we know exactly why you were like that… (Sandra, a 39*-*year*-*old female).*

In contrast, others perceived little emotional and social support from their loved ones. Participants perceived this lack of support to be as a consequence of family members’ limited understanding of their disorder. One participant recalled:

*You know I’ve explained to him […]I don’t think he understands why I get so anxious or why I, if I just cry, what it, he doesn’t see what’s going on in my head. Sometimes what’s going on in my head is so so much, that when you tell someone, that’s not much, you know but in your head it’s too big, so it’s very difficult to explain. […] I’m just saying that the level is different and yes he doesn’t always understand but he’s still there. Yes, he’s still there (Wendy, a 26*-*year*-*old female*).

## Discussion

In this study we qualitatively explored the ways in which OCD influences the daily life and functioning of South African adults living with OCD. We used EST as a broad theoretical lens through which to identify the composition of participant’s ecological systems. Our findings provide a closer look at participants’ microsystems, that is—the impact of OCD on the individual over time, on everyday living and relationships with family, peers and colleagues. Further, our findings also provide an account of the strategies participants used to cope with OCD, and the sources of support that were available to them.

Our study is part of a growing body of qualitative literature on OCD using exploratory approaches to better understand the perspectives of individuals living with OCD [11–13, 15, 17]. In her paper, Knapton [11] argues that the majority of studies on OCD are quantitative in nature and that our understanding of OCD, “has largely come from participants’ judgments of pre-defined and inflexible statements rather than from extended and unrestricted descriptions”, and that this approach limits, “intra-participant variation” (p. 2). In the current study, the findings provide instances of consistent *and* contrasting accounts of the experience of OCD—highlighting the heterogeneity of the disorder, and the importance of exploring individual perspectives.

Our findings provided an account of participant’s realisation of having OCD. Our findings demonstrated that, initially (often reflecting back to childhood), individuals with OCD perceived their symptoms as *normal*, that they were similar to other people, and that their behaviours and thoughts were *part of their personality*. It was only when symptoms began to markedly disrupt daily life and functioning, in addition to causing major distress, that they realised that these were pathological or indicative of having a psychiatric problem, warranting a visit to the clinic. Notably, research suggests that individuals with OCD sometimes lack such insight into the excessiveness of their symptoms [42]. Given that some individuals without OCD also experience *OC*-*like* symptoms, the behaviour is often interpreted as normal [43] or minimalized as “just a bad habit” that can easily be addressed. Family members and spouses in particular were key to helping participants recognise their symptoms as excessive. As shown in other research, participants’ compulsions i.e. externalised behaviour such as checking or washing, were key to recognition of the disorder [42]. However, it has been shown that family members themselves often experience marked distress when observing compulsions associated with OCD [16]. Interactions with significant others in the context of OCD was not necessarily perceived as positive though. For example, in their study of individuals living with OCD and their partners in Norway, Walseth et al. found that patients often reported feeling monitored and *surveillanced* by their partners [16]. This seems to be an important area for therapeutic focus, to eliminate potentially persecutory actions by family members towards those with OCD, and to also help mitigate the frustration and disconnect individuals with OCD feel with family members who seemingly do not understand them or their OCD [17]. Failure to address this may lead to avoidance of interaction by those with OCD and their partners and lead to performing behaviours in secrecy, and increasing what Walseth et al. [16] refer to as a ‘mental distance’.

Our findings also showcased the disruptions to daily life due to OCD. Similar to other studies [44, 45], our findings demonstrated OCD’s deep impact on sleep quality, leisure activities, and participants’ interpersonal relationships with friends and family. Participants in our study experienced difficulty preparing for sleep as thoughts and rituals had to be performed continuously. Evidence for sleep disruption due OCD is not consistent across studies, with some demonstrating difficulty sleeping and resulting exhaustion [46, 47], and others not [48]. The findings that do suggest sleep disturbance also included references to difficulties in daily functioning, overall physical and mental health [49]. For our participants, a lack of sleep and the associated consequences exacerbated their perceptions of their illness severity, ultimately affecting their ability to function daily [50]. Leisure activities are found to benefit and assist mental illness in the process of recovery [51]. However, consistent with other studies, participants found it difficult to engage in activities they enjoy, as many of these activities triggered symptoms of OCD. Moreover, engaging in leisure activities provide opportunities for socialisation. As such, withdrawal from these activities may ultimately also lead to isolation [45, 52].

Consistent with some quantitative [53, 54] and qualitative work [11, 13, 17], several participants commented on the impact of their OCD on attendance and performance at school/university, and/or work. In their study with nine British individuals living with OCD, the authors identified the theme of ‘Failing at life’ and described this as the overwhelming impact of the disorder on education and careers [13]. Similar to participants in our study, these participants spoke of their goals and expectations being delayed or thwarted as a consequence of this disorder [13]. Consequently, individuals with OCD may feel hopeless and powerless, because they cannot live up to particular expectations or potential, which impacts on their self-esteem [17]. On the other hand, there is not much evidence for the positive impact that OCD may have on school and work as found in this study, despite two qualitative studies making slight mention of it [10, 13]. For example, echoing the words of one of our participants, the one study [10] reported how OCD enabled one of their participants to be thorough and good with detail.

Our findings also showcased the ways in which participants managed the disruptions due to OCD in their lives. Participants identified physical, emotional and financial support from significant others as crucial in managing and coping with their OCD. This finding is consistent with studies that have shown the positive impact of perceived social support on individuals’ ability to cope with the difficulties of OCD [55, 56]. Similar to our findings, some studies show that most family members of individuals living with OCD accommodate them in most respects—e.g. actively assisting the OC individual by participating in their rituals or doing chores for them in order to save time or reduce frustration [16, 57]. However, there also is strong evidence to suggest that family support and involvement may exacerbate dysfunctional behaviours [58, 59]. Whereas participants in our study explicitly stated an appreciation of this type of accommodation, and perceived it as a means of social support—studies show that this type of accommodation seems to interfere with a positive response to treatment [16, 60]. Furthermore, while participants were mostly positive about the support they received from their significant others, there were concerns about their illness as being burdensome and traumatic for the families [61, 62]. Arguably, these concerns may lead to avoidance of sharing and ultimately, to feeling isolated. This hypothesis is consistent with findings from a recent qualitative study where participants reported feeling disconnected from family and friends [17]. As such, emotional responses towards family and friends may range from positive and appreciative to fears of being burdensome and withdrawal—highlighting that it is an important avenue for further research. Indeed, some literature suggests that involving family in treatment (e.g. partners, in couple-based therapy) may not be suitable for everyone [16]. Furthermore, other studies have also demonstrated that familial responses to OCD vary, and in some instances may become a source of conflict that can contribute to even poorer relationship functioning, marital discord, and divorce [63–65]. The inability to maintain a meaningful connection with others limits patients’ access to emotional support and is likely to perpetuate feelings of hopelessness and helplessness [10, 11, 13, 14, 17].

Participants in the current study experienced OCD as disturbing, destructive, and debilitating, causing strong emotional reactions such as anxiety, irritation, anger, sadness, discomfort, and disgust. This ties in with diagnostic nomenclatures such as the DSM [3] and the ICD [66], which describes OCD as a condition characterised by intrusive thoughts and compulsions associated with significant distress. Feelings of anger, dissatisfaction, and hopelessness are also typical of patients with mental health problems such as OCD [13, 17, 67]. Our participants reported various ways of coping and adjusting to, and being flexible about the demands of their condition and its sequelae. Participants reported shifting from first attempting to solve OCD itself (problem-focused coping) to attempting to solve the emotions associated with their OCD (emotion-focused coping) [68]. As participants shift from problem-focused coping to emotion-focused coping, they change their thoughts and the way they view their disorder and therefore move towards feelings of acceptance, i.e. accepting their condition and wanting to be accepted by others. This relative acceptance of an OCD diagnosis resonates with Relational Frame Theory (RFT) which forms the basis of Acceptance and Commitment Therapy (ACT) [69]. ACT is an empirically-based psychological intervention that uses acceptance strategies mixed with commitment and behavior-change strategies, to increase psychological flexibility. There is a fair amount of research on ACT as a model and a treatment for OCD spectrum disorders [70]. The acceptance/commitment concept leads us back to the present study’s first theme labelled “realisation of OCD”. As noted previously, whether participants accepted the diagnosis and its chronic nature or not, its impact on their daily lives is unmistakeable, and influences relationships significantly. It may be argued that if patients with OCD can learn to stop avoiding and denying their OC symptoms and accept the existence and the need for treatment thereof, they may be able to move forward in their lives. With this understanding, individuals may begin to accept their diagnosis and its sequelae, and commit to making the necessary changes in their behavior, regardless of what is going on in their lives.

## Limitations and recommendations

This study has a few limitations. First, the sample size is relatively small but nevertheless comparable to other qualitative work. Second, interviews were once-off, thus providing little opportunity for rapport building between the interviewer and the interviewee. Follow-up interviews may have provided an opportunity for participants to feel sufficiently more comfortable to provide more in-depth information. Finally, we acknowledge that interviews with family members, friends and work colleagues may have afforded a more in-depth account of the bi-directionality of the impact of OCD on members of participant’s microsystem. Interviews with these key role players in participants’ lives will be an important avenue for further research to go beyond just identifying individual level factors to target in interventions.

## Conclusion

In conclusion, this qualitative study of the lived experiences of adults with OCD identified 3 three main themes, centring on participants’ realisations of having OCD, the disruptions caused and the ways in which these were perceived and addressed. Our findings were consistent with those from quantitative studies, but provides added depth that may assist in the care of these patients. For example, in the current study the findings provide instances of consistent *and* contrasting accounts of the experience of OCD—highlighting the heterogeneity of the disorder, and the importance of exploring individual perspectives. The involvement of the family and significant others in the patient’s symptoms and treatment was also addressed; the findings here have emphasized that these relationships are dynamic and may not necessarily be constructive, warranting special attention and adjustment during therapy. A discussion of management and coping with OCD emphasized the importance of acceptance. This ties in with recent work on the potential value of ACT, either as monotherapy or as an adjunct to conventional techniques such as Cognitive Behavioural Therapy (CBT). Many caveats in our knowledge remain. For example, whether the South African experiences of living with OCD are significantly different from other contexts, is not yet known. Also, how local challenges—e.g. limited accessibility, affordability and specialization—shape experiences of South Africans with OCD, is another potential research avenue to be explored.
